# Supplementary material for: Regulation of RUVBL1-RUVBL2 AAA-ATPases by the nonsense-mediated mRNA decay factor DHX34, as evidenced by Cryo-EM
Source: eLife. 2020 Nov 18;9:e63042. doi: 10.7554/eLife.63042 (PMC7707835; doi:10.7554/eLife.63042)
Supplement: Figure 5—source data 1. — In each sheet is included: Name of sample, time (min), absorbance at 340 nm for the replicas, equation of the linear regression trendline for each replica used for data analysis, and R2 value of the linear regression trendline for each replica. [file elife-63042-fig5-data1.zip › Figure 5 - source data 1 explanations.docx]

Sheets related to Figure 5:

Sheet 1: Figure5_source_1: Measurements for sample His-RUVBL1-RUVBL2 from Figure 5 Panels A and B.

Sheet 2: Figure5_source_2: Measurements for sample His-RUVBL1-RUVBL2^E300Q^ from Figure 5 Panels A and C.

Sheet 3: Figure5_source_3: Measurements for sample His-RUVBL1^E303Q^-RUVBL2 from Figure 5 Panels A and C.

Sheet 4: Figure5_source_4: Measurements for sample His-RUVBL1^E303Q^-RUVBL2^E300Q^ from Figure 5 Panel A.

Sheet 5: Figure5_source_5: Measurements for sample His-RUVBL1-RUVBL2 + DHX34^D279A^ from Figure 5 Panel B.

Sheet 6: Figure5_source_6: Measurements for sample His-RUVBL1-RUVBL2^E300Q^ + DHX34^D279A^ from Figure 5 Panel C.

Sheet 7: Figure5_source_7: Measurements for sample His-RUVBL1^E303Q^-RUVBL2 + DHX34^D279A^ from Figure 5 Panel C.

- Graphic in Figure 5 Panel A: plotting of the average of ATP hydrolysis indicated as percentage for His-RUVBL1-RUVBL2 (Figure5_source_1), His-RUVBL1-RUVBL2^E300Q^ (Figure5_source_2), His-RUVBL1^E303Q^-RUVBL2 (Figure5_source_3) and His-RUVBL1^E303Q^-RUVBL2^E300Q^ (Figure5_ source_4).

- Graphic in Figure 5 Panel B: plotting of the average of ATP hydrolysis indicated as percentage for His-RUVBL1-RUVBL2 (Figure5_source_1) and His-RUVBL1-RUVBL2 + DHX34^D279A^ (Figure5_source_5).

- Graphic in Figure 5 Panel C: plotting of the average of ATP hydrolysis indicated as percentage for His-RUVBL1-RUVBL2^E300Q^ (Figure5_source_2), His-RUVBL1-RUVBL2^E300Q^ + DHX34^D279A^ (Figure5_source_6), His-RUVBL1^E303Q^-RUVBL2 (Figure5_source_3) and His-RUVBL1^E303Q^-RUVBL2 + DHX34^D279A^ (Figure5_source_7).

Sheets related to Figure 5 – figure supplement 1:

Sheet 8: Figure5_source_8: Measurements for sample DHX34 from Figure 5 – figure supplement 1 Panels D and E.

Sheet 9: Figure5_source_9: Measurements for sample DHX34^D279A^ from Figure 5 – figure supplement 1 Panels D and E.

- Graphic in Figure 5 – figure supplement 1 Panel D: plotting of replica 1 from Figure5_source_8 (DHX34) and replica 2 from Figure5_source_9 (DHX34 ^D279A^).

- Graphic in Figure 5 – figure supplement 1 Panel D: plotting of the average of ATP consumption indicated as percentage for DHX34 (Figure5_source_8) and DHX34^D279A^ (Figure5_source_9).

Graphics in Figure 5 – figure supplement 2:

- Graphic in Figure 5 – figure supplement 2 Panel A: plotting of replica 4 from Figure5_source_1 (His-RUVBL1-RUVBL2), replica 3 from Figure5_source_2 (His-RUVBL1-RUVBL2^E300Q^), replica 1 from Figure5_source_3 (His-RUVBL1^E303Q^-RUVBL2) and replica 1 from Figure5_source_4 (His-RUVBL1^E303Q^-RUVBL2 ^E300Q^).

- Graphic in Figure 5 – figure supplement 2 Panel B: plotting of replica 4 from Figure5_source_1 (His-RUVBL1-RUVBL2) and replica 3 from Figure5_source_5 (His-RUVBL1-RUVBL2 + DHX34^D279A^).

- Graphic in Figure 5 – figure supplement 2 Panel C: plotting of replica 3 from Figure5_source_2 (His-RUVBL1-RUVBL2^E300Q^) and replica 2 from Figure5_source_6 (His-RUVBL1-RUVBL2^E300Q^ + DHX34^D279A^).

- Graphic in Figure 5 – figure supplement 2 Panel D: plotting of replica 1 from Figure5_source_3 (His-RUVBL1^E303Q^-RUVBL2) and replica 1 from Figure5_source_7 (His-RUVBL1^E303Q^-RUVBL2 + DHX34^D279A^).

Sheets related to Figure 5 – figure supplement 3:

Sheet 10: Figure5_S3_source_10: Measurements for sample Untagged RUVBL1-RUVBL2 from Figure 5 – figure supplement 3 Panels A, B, C and D.

Sheet 11: Figure5_S3_source_11: Measurements for sample Untagged RUVBL1-RUVBL2 + DHX34^D279A^ from Figure 5 – figure supplement 3 Panels B and D.

Sheet 12: Figure5_S3_source_12: Measurements for sample Untagged RUVBL1-RUVBL2^E300Q^ from Figure 5 – figure supplement 3 Panels E and G.

Sheet 13: Figure5_S3_source_13: Measurements for sample Untagged RUVBL1-RUVBL2^E300Q^ + DHX34^D279A^ from Figure 5 – figure supplement 3 Panels E and G.

Sheet 14: Figure5_S3_source_14: Measurements for sample Untagged RUVBL1^E303Q^-RUVBL2 from Figure 5 – figure supplement 3 Panels F and G.

Sheet 15: Figure5_S3_source_15: Measurements for sample Untagged RUVBL1^E303Q^-RUVBL2 + DHX34^D279A^ from Figure 5 – figure supplement 3 Panels F and G.

- Graphic in Figure 5 – figure supplement 3 Panel A: plotting of replica 4 from Figure5_source_1 (His-RUVBL1-RUVBL2) and replica 1 from Figure5_S3_source_10 (Untagged RUVBL1-RUVBL2).

- Graphic in Figure 5 – figure supplement 3 Panel A: plotting of the average of ATP consumption indicated as percentage for His-RUVBL1-RUVBL2 (Figure5_source_1) and Untagged RUVBL1-RUVBL2 (Figure5_S3_source_10).

- Graphic in Figure 5 – figure supplement 3 Panel C: plotting of replica 1 from Figure5_S3_source_10 (Untagged RUVBL1-RUVBL2) and replica 1 from Figure5_S3_source_11 (Untagged RUVBL1-RUVBL2 + DHX34^D279A^).

- Graphic in Figure 5 – figure supplement 3 Panel D: plotting of the average of ATP consumption indicated as percentage for Untagged RUVBL1-RUVBL2 (Figure5_S3_source_10) and Untagged RUVBL1-RUVBL2 + DHX34^D279A^ (Figure5_S3_source_11).

- Graphic in Figure 5 – figure supplement 3 Panel E: plotting of replica 1 from Figure5_S3_source_12 (Untagged RUVBL1-RUVBL2^E300Q^) and replica 2 from Figure5_S3_source_13 (Untagged RUVBL1-RUVBL2^E300Q^ + DHX34^D279A^).

- Graphic in Figure 5 – figure supplement 3 Panel F: plotting of replica 3 from Figure5_S3_source_14 (Untagged RUVBL1^E303Q^-RUVBL2) and replica 1 from Figure5_S3_source_15 (Untagged RUVBL1^E303Q^-RUVBL2 + DHX34^D279A^).

- Graphic in Figure 5 – figure supplement 3 Panel G: plotting of the average of ATP consumption indicated as percentage for Untagged RUVBL1-RUVBL2^E300Q^ (Figure5_S3_source_12), Untagged RUVBL1-RUVBL2^E300Q^ + DHX34^D279A^ (Figure5_S3_source_13), Untagged RUVBL1^E303Q^-RUVBL2 (Figure5_S3_source_14) and Untagged RUVBL1^E303Q^-RUVBL2 + DHX34^D279A^ (Figure5_S3_source_15).
